# Editorial: Contextual inflammation: mechanisms of immune adaptation across tissues, time, and disease states

**DOI:** 10.3389/fimmu.2026.1987081

**Published:** 2026-09-17

**Authors:** Amro M. Soliman, Paulo J. Basso, Silvana Morello

**Affiliations:** 1 Department of Biological Sciences, Concordia University of Edmonton, Edmonton, AB, Canada; 2 Department of Medical Microbiology and Immunology, University of Alberta, Edmonton, AB, Canada; 3 Department of Pharmacy, University of Salerno, Fisciano (SA), Italy

**Keywords:** immune dysregulation, immunometabolism, immunomodulation, inflammation, microbiota-immune axis

The inflammatory process protects organisms against environmental challenges and contributes to the maintenance of physiological homeostasis (1). Inflammation is triggered by diverse stimuli, including infections and tissue injury, and involves a coordinated, multistep response aimed at eliminating the underlying source of disturbance (2). The process requires activation of immune and tissue-resident cells, accompanied by the production and release of proinflammatory cytokines (3). These mediators act through both autocrine and paracrine mechanisms to promote changes in vascular permeability, facilitating recruitment and positioning of additional immune cells at sites of inflammation, thereby supporting the elimination of invading pathogens. Although inflammation is fundamentally a protective mechanism, dysregulation or inappropriate persistence of the inflammatory response can result in pathological consequences (4), including cardiovascular and autoimmune disorders, infectious diseases and cancer (5). The clinical effectiveness of therapies that selectively target inflammatory pathways further highlights the importance of elucidating the mechanisms that initiate, regulate and resolve inflammation.

Inflammation is increasingly understood not as a uniform or linear immune response, but as a highly dynamic process shaped by tissue environment, cellular composition, metabolic state, microbial signals, temporal cues and disease context. The outcome of an inflammatory response (i.e., resolution, persistence, tissue injury, or immune suppression) depends on the interaction of these factors across multiple spatial and temporal scales. It is critical to understand how inflammatory responses are initiated, adapted, sustained and resolved while recognizing three interconnected principles:

1. inflammation is spatially organized (tissue-dependent),
2. temporally adaptive,
3. and metabolically and systemically integrated.

The tissue context is crucial in determining the features of inflammatory responses. For example, in the liver during *Staphylococcus aureus* bloodstream infection, hepatocytes are active participants in the early immune response, with their responses differing according to their position along the hepatic porto-central axis. Periportal and midzonal hepatocytes exhibited stronger transcriptional activation than pericentral hepatocytes, highlighting how tissue architecture can establish distinct immune programs within the same organ (Nwofor et al.). Similarly, pregnane X receptor (NR1I2/PXR), a key regulator of inflammation and intestinal homeostasis, has been shown to have effects that are dependent on tissue type in addition to other ligand and pathological context (Amini). Rather than functioning simply as an anti-inflammatory switch, NR1I2 also integrates xenobiotic metabolism, microbial-derived signals, intestinal barrier function and innate immune regulation (Amini). These observations reinforce the importance of understanding inflammatory regulators within their specific tissue and physiological environments.

The temporal dimension represents another cornerstone in our understanding of inflammation. A key example is provided by macrophage responses during prolonged TLR4 stimulation (Mazzio et al.). Transcriptomic analysis reveals a transition from acute activation toward an active, immunosuppressive state during chronic stimulation, characterized by increased regulatory and checkpoint-associated programs as well as reduced antigen-presentation and interferon-response signatures (Mazzio et al.). Temporal adaptation of inflammatory responses can also be influenced by biological sex and tissue context. Rito-Fernandes et al. examined peripheral and local immune responses following spinal cord injury and found sex-dependent differences that varied across acute and chronic stages of injury. During the acute phase, males showed higher frequencies of circulating myeloid cells, whereas females exhibited greater myeloid cell accumulation within the injured spinal cord, together with distinct inflammatory and reparative-associated features. These differences were less pronounced at later stages, highlighting how immune responses can change over time and differ between peripheral and tissue compartments. This concept of inflammatory memory extends beyond immune cells to other cell types. Voli et al. demonstrate that a transient inflammatory stimulus can produce durable changes in gastric epithelial cells. Brief exposure to IFNγ resulted in persistent repression of the tumor suppressor TFF1 through transcriptional and epigenetic mechanisms (Voli et al.). These findings highlight how inflammatory signals can leave long-lasting molecular imprints in non-immune cells, potentially linking transient inflammation to chronic tissue dysfunction and carcinogenesis.

Interestingly, regulation of the inflammatory response is further inseparable from cellular metabolism and systemic physiology. An integrated metabolism–immune–neuro framework for post-exertional malaise has been proposed, linking mitochondrial dysfunction, oxidative stress, immune activation and neuroinflammation in a self-reinforcing cycle (Jin et al.). This perspective illustrates how inflammatory processes may extend beyond individual tissues and become embedded within broader physiological networks. A related systems-level perspective, the Bio-Intelligence Circuit framework, was proposed linking mitochondrial stress, innate immune signalling, nuclear receptor regulation and autonomic control (Subramaniyan and Parthiban). The circuit indicates how interactions among metabolic, immune and neural regulatory systems determine whether inflammatory states remain persistent or become permissive to resolution. Likewise, the interaction between the nervous system, microbiota, metabolism and local inflammatory signalling can determine the outcome of an inflammatory reaction, as demonstrated in a functional dyspepsia model (Zhang et al.). Researchers showed that electroacupuncture altered vagal activity, duodenal microbiota, metabolites and therefore inflammatory pathways (Zhang et al.). These findings underscore the extent to which neuroimmune and microbiota–host interactions can influence tissue-specific inflammation. Remarkably, circadian regulation can also influence immune-cell recruitment, activation, resolution and tissue remodelling during cutaneous wound healing (Luo et al.).

In addition to the previously mentioned levels of regulation, several specific molecular and cellular mechanisms can determine inflammatory outcomes. For instance, conventional dendritic cells, particularly cDC1 cells, have been identified as prominent transcriptionally perturbed populations in human sepsis. They have been shown to implicate ubiquitination-associated pathways, including UBE2F, in inflammatory dysregulation (Li et al.). Another mechanism through which inflammation and collateral tissue damage intersect is PANoptosis, an integrated inflammatory cell death pathway incorporating features of pyroptosis, apoptosis, and necroptosis. Current evidence indicates that PANoptosis integrates multiple cell-fate signalling pathways and interconnected molecular mechanisms, generating a complex inflammatory network that can exacerbate tissue injury and organ dysfunction (Chen et al.).

Taken together, the biological meaning of inflammation depends on where it occurs, when it occurs, which cells participate, and the metabolic and physiological environment in which it unfolds. Tissue architecture, cellular plasticity, inflammatory memory, metabolism, microbiota, circadian rhythms, and neuroimmune communication collectively determine whether inflammation promotes host defense and repair or progresses toward chronic dysfunction. This contextual view has important implications for therapeutic development. Rather than broadly suppressing inflammation, future strategies may need to identify the specific cellular and tissue programs that sustain pathological inflammation while preserving protective immune functions. Defining these context-specific mechanisms may ultimately enable precise interventions that selectively interrupt pathological inflammation, restore resolution and promote tissue repair.
